# Technical challenges of modelling real-life epidemics and examples of overcoming these

**DOI:** 10.1098/rsta.2022.0179

**Published:** 2022-10-03

**Authors:** J. Panovska-Griffiths, W. Waites, G. J. Ackland

**Affiliations:** ^1^ The Big Data Institute and the Pandemic Sciences Institute, Nuffield Department of Medicine, University of Oxford, Oxford, UK; ^2^ The Queen’s College, University of Oxford, Oxford, UK; ^3^ Department of Computer and Information Sciences, University of Strathclyde, Glasgow G1 1XH, UK; ^4^ Institute of Condensed Matter and Complex Systems, School of Physics and Astronomy, University of Edinburgh, Edinburgh EH9 3FD, UK

**Keywords:** epidemics, mathematical modelling, COVID-19

## Abstract

The coronavirus disease 2019 (COVID-19) pandemic has highlighted the importance of mathematical modelling in informing and advising policy decision-making. Effective practice of mathematical modelling has challenges. These can be around the technical modelling framework and how different techniques are combined, the appropriate use of mathematical formalisms or computational languages to accurately capture the intended mechanism or process being studied, in transparency and robustness of models and numerical code, in simulating the appropriate scenarios via explicitly identifying underlying assumptions about the process in nature and simplifying approximations to facilitate modelling, in correctly quantifying the uncertainty of the model parameters and projections, in taking into account the variable quality of data sources, and applying established software engineering practices to avoid duplication of effort and ensure reproducibility of numerical results. Via a collection of 16 technical papers, this special issue aims to address some of these challenges alongside showcasing the usefulness of modelling as applied in this pandemic.

This article is part of the theme issue ‘Technical challenges of modelling real-life epidemics and examples of overcoming these’.

Since the onset of the COVID-19 pandemic, in a number of countries, including the UK, mathematical and statistical modelling has been at the forefront of informing the status of the epidemic and providing evidence around possible future scenarios to decision makers. Modelling gives a flexible theoretical framework which, parameterized with and calibrated to data, allows exploration of different transmission scenarios and evaluation of different interventions. Although mathematical modelling of infectious diseases has a long history, this pandemic highlighted its applicability and importance for real-time decision-making. The pandemic has also driven the development and application of novel mathematical and computational methods, some of which are presented in this special issue. Undertaking real-time modelling is challenging as it requires a trade-off between fast and less robust modelling and delayed but more robust modelling. The former can be appropriate when data is scarce and we know very little, like at the onset of the pandemic, or when new variants of the virus occur. The latter can be appropriate when we are faced with an already developed epidemic and changes in response may be necessary; for example when faced with a fragmented epidemic where the classic measures such as national reproduction number R values may not be appropriate or when developing and rolling out a vaccination strategy.

During the current pandemic, most of the modelling groups involved in advising the UK Government have been undertaking responsive and real-time modelling, often balancing the need to give quick analysis outcomes with assuring accuracy and uncertainty quantification. While undertaking and publishing timely modelling results is important to advice on the current pandemic, as part of the lessons learned from this pandemic and in preparation for the next, it is important that modellers invest in testing the rigorousness in deriving outcomes. Real-time modelling and the vast amount of models developed in the last 24 months have highlighted gaps in the existing technical frameworks that ought to be addressed in preparedness for possible future pandemics.

Much of the work in this edition was supported by the Royal Society Rapid Assistance in Modelling the Pandemic [1] initiative which mobilized modelling expertise from a diverse range of disciplines to support the pandemic modelling community already working on Coronavirus (COVID-19). Details of the initiative, its origin, purpose, aims and outcomes are described in a separate article within this edition [2].

This special issue showcases a combination of articles that address overarching technical issues identified in the course of the current epidemic that would be relevant when modelling future ones. Through a series of articles, we generate a library of tools that promote open and transparent science and would be relevant as pandemic outbreak analysis tools in future.

Although each of the articles within the collections makes a fruitful singular contribution to scientific modelling, there are themes that link them. Figure 1 illustrates one possible way of classifying the articles in this issue according to subject matter. Most articles are concerned with more than one of these concepts and the edges show this. For example, the article by Marion *et al.* [3] presents an improved version of the approximate Bayesian computation algorithm and shows how this can be used to fit to data. The article by Moore *et al.* [4] on the use of scoring rules to refine forecasts sits in a similar conceptual space.

**Figure 1.**
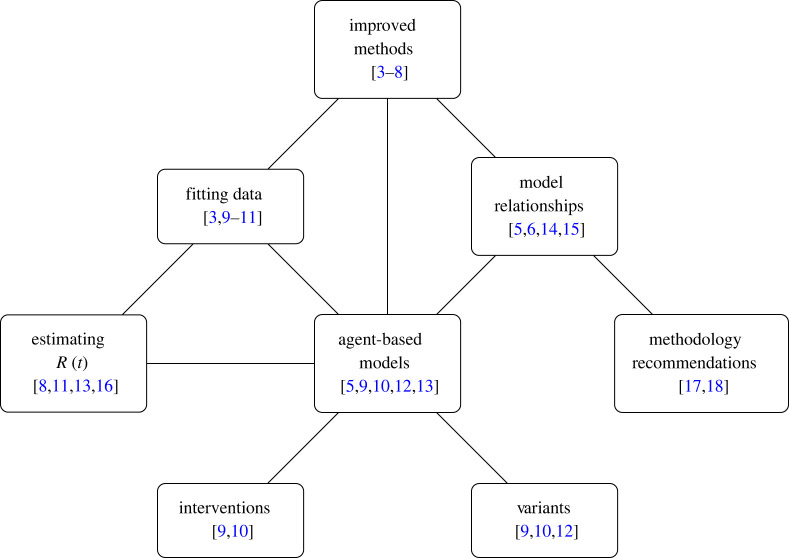
Conceptual map of the articles in this issue. Each article fits into at least one of these subject areas. Where an article treats more than one subject, an edge is drawn between them.

Agent-based models (ABMs) figure prominently in this issue with [5–8] all using them to explore different questions. All of these involve fitting to real data, of course, and all consider the effect of different variants of SARS-CoV-2. Sanz-Leon *et al.* [7] investigate vaccine hesitancy and the δ variant in Australia. Panovska-Griffiths *et al.* [5] and Hinch *et al.* [6] are focused on the English COVID-19 epidemics and both estimate the transmissibility of variants and evaluate interventions, with the former combining the ABM with a statistical regression and making a policy contribution with the results, while the latter is taking a geospatial approach.

A measure that has been important for policy decision-making and public understanding of the pandemic is the basic reproduction number, $R_{0}$, and its time-varying analogue, effective reproduction number, R(t). Ackland *et al.* [9] show how to estimate this function in the context of UK data and explains why it behaves as it does. Li *et al.* [8] use an ABM to estimate R(t) and demonstrate improved statistical methods to do this in the face of noisy data, while Creswell *et al.* [10] use classic modelling techniques to explore the role of imported COVID-19 cases on R(t). Finally, Madzvamuse *et al.* [11] present analytical results showing how to reformulate the standard differential equation epidemic model so that it is expressed in terms of observed information rather than the unobserved actual state of the population.

Complementing the foregoing, which are concerned with particular models, are several papers that consider relationships between models and borrow methods from other aspects of mathematics to answer epidemiological questions. Fairbanks *et al.* [12] show how the mathematical property of compositionality can be used to construct complex compartmental models out of simpler ones in a semi-automated, modular, and type-safe way. Waites *et al.* [13] also demonstrate how the compositional and modular properties of rule-based models—used widely in physics—can be used to show how a simple immune response model can produce the heterogeneity that we observe at the population level. Probert *et al.* [14] also used rule-based methods—this time borrowed from vote-processing analogy—to explore the uncertainty of combining models’ outcomes. Vernon *et al.* [15] show how simpler ‘emulators’ models can simulate more complex ABMs to assist in parameter estimation and sensitivity analysis. Swallow *et al.* [16] borrow methodology from statistics and use principal component analysis to derive key epidemic metrics across the four UK nations.

Finally, two papers take a high-level view and make recommendations about modelling in general. Mitchell *et al.* [17] emphasize the importance of provenance, describing a pipeline for managing data and promoting a practice of transparency and reproducibility in scientific workflows that should be adopted in all modelling endeavours. Chen *et al.* [18] tackle visualization and make recommendations for the challenges surrounding communication of results.

In summary, this special issue addresses a number of questions around modelling that have not been considered in the rapid responsive modelling of the ongoing epidemics. Via a collection of papers that include multiscale, cross-discipline mathematical and statistical modelling, and focus on technical aspects of modelling rather than solely applications, while being timely and relevant to the current pandemic, this special issue aims to withstand time and be relevant for possible future pandemics also.

## Data Availability

This article has no additional data.
